# Methacryloyl-GlcNAc Derivatives Copolymerized with Dimethacrylamide as a Novel Antibacterial and Biocompatible Coating

**DOI:** 10.3390/pharmaceutics13101647

**Published:** 2021-10-09

**Authors:** Max Borgolte, Oliver Riester, Tereza Kacerova, Simone Rentschler, Magnus S. Schmidt, Susanne Jacksch, Markus Egert, Stefan Laufer, René Csuk, Hans-Peter Deigner

**Affiliations:** 1 Institute of Precision Medicine, Furtwangen University, Jakob-Kienzle Str. 17, 78054 Villingen-Schwenningen, Germany; Max.Borgolte@hs-furtwangen.de (M.B.); Oliver.Riester@hs-furtwangen.de (O.R.); S.Rentschler@hs-furtwangen.de (S.R.); Magnus.Schmidt@hs-furtwangen.de (M.S.S.); Susanne.Jacksch@hs-furtwangen.de (S.J.); Markus.Egert@hs-furtwangen.de (M.E.); 2 Department of Organic Chemistry, Martin-Luther University Halle-Wittenberg, Kurt-Mothes Str. 2, 06120 Halle (Saale), Germany; Rene.csuk@chemie.uni-halle.de; 3 Department of Pharmaceutical and Medicinal Chemistry, Institute of Pharmaceutical Sciences, Eberhard Karls University Tuebingen, Auf der Morgenstelle 8, 72076 Tübingen, Germany; Stefan.laufer@uni-tuebingen.de; 4 Faculty of Science, Eberhard Karls University Tuebingen, Auf der Morgenstelle 8, 72076 Tübingen, Germany; 5 Department of Chemistry, Czech University of Life Sciences, Kamýcká 129, 16500 Prague, Czech Republic; tereza.kacerova.18@ucl.ac.uk; 6 Department of Chemistry, University College London, London WC1H 0AJ, UK; 7 EXIM Department, Fraunhofer Institute IZI (Leipzig), Schillingallee 68, 18057 Rostock, Germany

**Keywords:** carbohydrates, glycosides, antibacterial, antibiofilm, MRSA, *E. coli*, biocompatible

## Abstract

Improving medical implants with functional polymer coatings is an effective way to further improve the level of medical care. Antibacterial and biofilm-preventing properties are particularly desirable in the area of wound healing, since there is a generally high risk of infection, often with a chronic course in the case of biofilm formation. To prevent this we here report a polymeric design of polymer-bound *N*-acetyl-glucosamine-oligoethylene glycol residues that mimic a cationic, antibacterial, and biocompatible chitosan surface. The combination of easy to use, crosslinkable, thin, potentially 3D-printable polymethacrylate layering with antibacterial and biocompatible functional components will be particularly advantageous in the medical field to support a wide range of implants as well as wound dressings. Different polymers containing a *N*-acetylglucosamine-methacryloyl residue with oligoethylene glycol linkers and a methacryloyl benzophenone crosslinker were synthesized by free radical polymerization. The functional monomers and corresponding polymers were characterized by ^1^H, ^13^C NMR, and infrared (IR) spectroscopy. The polymers showed no cytotoxic or antiadhesive effects on fibroblasts as demonstrated by extract and direct contact cell culture methods. Biofilm formation was reduced by up to 70% and antibacterial growth by 1.2 log, particularly for the 5% GlcNAc-4EG polymer, as observed for Escherichia coli and Staphylococcus aureus as clinically relevant Gram-negative and Gram-positive model pathogens.

## 1. Introduction

Bacterial wound infections are a major health problem, comparable to infections subsequent to surgical procedures, especially when a biofilm is formed significantly reducing the susceptibility of bacteria to antibiotics [1,2,3]. In combination with the increasing number of reported multidrug-resistant pathogens, antibiotic resistant bacterial infections are a clinical problem that will become even more acute in the future [4]. An implant or scaffold has to be functional for fibroblast or stem cells adhesion to ensure proper resorption of the implant into the surrounding tissue. This functionalization promotes adhesion often unspecifically for all cells and organisms, including bacteria, leading to a “race to the surface” [5,6], whereby the patient’s cells and bacteria compete to adhere to an implant’s surface. The critical time window for this competition between body tissue and bacteria has been determined to be the first 6 h after implantation, while a single bacteria can form a biofilm within 24 h [7]. After 48–96 h, the biofilm becomes resistant to therapeutic treatment as the formed matrix renders the encapsulated bacteria less susceptible to host defense mechanisms and antibiotic therapy [8,9,10,11,12]. Conventionally, antibiotic prophylaxis is used in both implant surgery and traumatic wound care to reduce the likelihood of bacterial infections [13,14]. However, the problem of increasing multidrug-resistant bacteria, particularly in clinical settings, has led to a re-evaluation of the extensive use of antibiotics [15,16]. To some degree, reducing the usage of systemic antibiotics can prevent the emergence of new multidrug-resistant pathogens. In order to prevent infections even with reduced administration of antibiotics, other antimicrobial mechanisms must also be applied, such as antimicrobial modification of surfaces or addition of nanoparticles [17,18,19].

Cationic polymers have been widely described in articles and reviews with regard to their antibacterial properties and use in self-disinfecting surfaces; most contain a quaternary ammonium group or alkyl pyridinium group as the functional component [20,21,22,23,24,25]. The mechanism of action of these cationic polymers in solution is well described by the Shai-Matsuzaki-Huang (SMH) model [26,27,28]. The antibacterial action of surfaces coated with cationic polymers is thought to follow a similar mechanism through polymeric brushes [21,29,30,31,32,33], but some publications describe a simple monolayer of cationic groups as being antibacterial as well [34]. Murata et al. propose a mechanism driven mainly by surface charge instead of insertion of cationic polymer brushes into the bacterial cell wall, following an SMH-like mechanism [20]. The exact mechanism of the antibacterial effect of cationic polymers requires further discussion and clarification.

Chitosan is a cationic polymeric aminoglycan, consisting of *N*-acetylglucosamine (GlcNAc) and glucosamine repeating units; it is used in tissue engineering applications such as bone tissue engineering [35,36,37], stem cell encapsulation [38,39,40], and wound dressing [41,42]. The polymer is thought to exhibit its antibacterial properties through a cationic mechanism via glucosamine’s amino group by disrupting the outer and inner bacterial cell membrane [43,44,45] and has been shown to mediate biofilm formation of *Actinobacillus pleuromoniae* [46]. The corresponding monomer, GlcNAc, has been shown to prevent biofilm formation by *Escherichia coli* [47]. Because of its insolubility in water and organic solvents, except ionic liquids, chitosan has been used in several polymeric modifications to combine its proliferative and antibacterial properties with the mechanical stiffness of other polymers, taking advantage of different synthetic polymers or nanoparticles, rendering it useful for biomedical applications [48].

Another group of antimicrobial compounds is the 1,2,3-triazoles, which exhibit antibacterial activity mainly through formation of hydrogen bonds of the triazole ring with other moieties, forming a cationic surface and possibly leading to an SMH-like antibacterial mechanism [49]. These 1,2,3-triazoles can easily be introduced by Huisgen 1,3-dipolar cycloaddition of an azide and alkyne, without the need for complicated workup procedures or toxic and expensive reagents, making it suitable for polymer modifications [50,51]. Several polymers modified in this way are listed in a library of (1,2,3-triazol-1-yl)quinazolin-4-ones and have shown antibacterial properties against Gram-positive and Gram-negative bacteria [52,53]. In addition, a library of 1,2,3-triazol-sucrose derivatives showed antifungal and antibacterial properties while maintaining low cytotoxicity against non-tumor cell lines [54,55,56].

An additional approach is the design of antiadhesive surfaces to prevent colonization of implant surfaces. Pandiyarajan et al. [57] described a surface-attached hydrogel network, consisting of poly-dimethacrylamide copolymerized with methacryloyl benzophenone (MBP), that had antiadhesive properties against proteins and blood platelets. Surface anchoring was accomplished via photoactive UV crosslinking of the benzophenone moiety [57]. The benzophenone undergoes a UV-induced, radical C–H insertion reaction, as reviewed by Prucker et al. [58] rendering it suitable for functionalization of polymeric surfaces to obtain stable, covalently attached hydrogel networks [58]. To the best of our knowledge, there is no description of combining the antimicrobial properties of a cationic GlcNAc residue with an antiadhesive, UV-crosslinkable acrylamide hydrogel in order to reduce microbial contamination of implant surfaces in the literature.

In this study, we combined the previously described approaches that resulted in a surface-bound poly-dimethacrylamide methyl (PDMAm) network with UV-induced anchoring via benzophenone, combining it with a GlcNAc residue containing a triazole and a distinct linker to the PDMAm network backbone. We thereby mimicked an antimicrobial chitosan surface while taking advantage of the antiadhesive PDMAm hydrogel as base layer. The hydrogel network can be easily obtained via solvent casting of the polymer solution, followed by UV crosslinking. We investigated the effect of the combinatorial approach of triazole functional groups and chitosan-mimicking surfaces on the antimicrobial, antibiofilm, and biocompatible properties of such coatings.

## 2. Experimental Section

### 2.1. Chemical Synthesis and Characterization

#### 2.1.1. General Methods

TLC was carried out on Silica Gel 60 F254 (Merck KGaA, Darmstadt, Germany, layer thickness 0.2 mm) with detection by UV light (254 nm) or by charring with 1% KMnO_4_ in 1N NaOH. Flash column chromatography (FC) was performed on M&N Silica Gel 60 (0.063–0.200 mm, MACHEREY-NAGEL GmbH, Düren, Germany). ^1^H NMR and ^13^C NMR spectra were recorded on a Bruker Avance I 200, Bruker Avance II 400 (Bruker Corporation, Billerica, MA, USA), or Varian Unity 500 (Varian, Palo Alto, CA, USA) spectrometer. Chemical shifts are reported in parts per million relative to solvent signals (CDCl_3_: δH = 7.26 ppm, δC = 77.0 ppm; DMSO-d6: δH = 2.49 ppm, δC = 39.7 ppm; CD_3_OD: δH = 4.78 ppm, δC = 49.3 ppm). Signals were assigned by first-order analysis, and assignments were supported, where feasible, by 2-dimensional ^1^H, ^1^H and ^1^H, ^13^C correlation spectroscopy. Coupling constants are reported in hertz. Chemicals and reagents were purchased from Acros Organics (Geel, Belgium), Sigma-Aldrich (Munich, Germany), Carl Roth (Karlsruhe, Germany), ABCR (Karlsruhe, Germany), or MCAT (Donaueschingen, Germany) and were used without further purification.

#### 2.1.2. Synthesis of Azido Linkers **2, 4, 5, 6**

Azido linkers were synthesized according to a procedure published by Mahou and Wandrey [59], following a cascade of sequential tosylation and NaN_3_ substitution steps. For diethylene glycol linker **2**, 2-(2-chloro-ethoxy)-ethanol was chosen as the starting material instead of the tosylated diethylene glycol residue, according to another published procedure [60].

#### 2.1.3. General Tosylation Procedure

Tosylation was carried out according to a literature report [59]. The corresponding linker (1 eq) and *p*-toluenesulfonyl chloride (1.1 eq) were dissolved in dichloromethane (DCM) at 0 °C and NEt_3_ (2 eq) was added. After stirring for 2 h, the ice bath was removed and the mixture stirred overnight at room temperature. Washing twice with water and once with brine, followed by evaporation of the solvent, yielded the tosylated linker as a yellowish oil.

#### 2.1.4. Chain Prolongment of Azido Linkers via Tosylate **5, 6**

Chain prolongment was carried out according to the literature [59]. Tosylated azido linker **4** (1 eq) and NaH (1.1 eq) were suspended in water-free tetrahydrofuran (THF) under Ar atmosphere. After stirring for 30 min at room temperature, diethylene glycol for product **5** or 1,8-octanediol for product **6** (5 eq) was added dropwise to the mixture. After stirring for 48 h under reflux, H_2_O was added, the solvent evaporated, and the aqueous layer extracted 3× with DCM Washing 2× with NaOH, followed by evaporation of the solvent and column chromatography (ethyl acetate/methanol 19:1, R_f_ = 0.4), yielded the pure products **5** and **6** as yellowish oils.

##### 8-(2-{2-[2-(2-Azido-ethoxy)-ethoxy]-ethoxy}-ethoxy)-octan-1-ol **6**

Yield: 71%

^1^H-NMR (CDCl_3_, 400 MHz): 3.71–3.63 (m, 14H, O–CH_2_), 3.59 (dt, *J* = 4.5, 1.2 Hz, 2H, O–CH_2_), 3.46 (t, *J* = 6.8 Hz, 2H, O–CH_2_), 3.40 (t, *J* = 5.1 Hz, 2H, N_3_–CH_2_), 1.57 (dt, *J* = 13.2, 6.5 Hz, 4H, CH_2_), 1.40–1.29 (m, *J* = 17.9 Hz, 8H, CH_2_).

^13^C-NMR (CDCl_3_, 100 MHz): 71.48 (O–CH_2_), 70.72 (O–CH_2_), 70.70 (O–CH_2_), 70.65 (O–CH_2_), 70.61 (O–CH_2_), 70.08 (O–CH_2_), 70.04 (O–CH_2_), 63.01 (O–CH_2_), 50.70 (N_3_–CH_2_), 32.77 (CH_2_), 29.60 (CH_2_), 29.39 (CH_2_), 29.33 (CH_2_), 26.00 (CH_2_), 25.66 (CH_2_).

#### 2.1.5. General Procedure for the Synthesis of Azidomethacrylates **7–10**

Azido methacrylates were synthesized as published [61]. Briefly, azido linker (1 eq) and NEt_3_ (1.3 eq) were dissolved in water-free DCM in a sealed Schlenk flask under Ar atmosphere cooled in an ice bath to 0 °C. Methacryloyl chloride (1.2 eq) was added dropwise to the mixture. The solution was allowed to warm to room temperature and stirred at room temperature overnight. Washing with 1M H_2_SO_4_ (3×, equal volume to solvent) followed by drying over Na_2_SO_4_ and evaporation of the solvent yielded the crude product. Further purification by column chromatography (ethyl acetate/hexane 1:10) yielded the pure products.

##### 2-Methyl-acrylic acid 2-(2-azido-ethoxy)-ethyl ester **7**

Yield: 81%

^1^H-NMR (CDCl_3_, 400 MHz): 6.17 (s, 1H, CH_2_), 5.60 (s, 1H, CH_2_), 4.34 (t, *J* = 4.7 Hz, 2H, O–CH_2_), 3.78 (t, *J* = 4.7, 2H, O–CH_2_), 3.71 (t, *J* = 5.0, 2H, O–CH_2_), 3.40 (t, *J* = 4.9, 2H, CH_2_–N_3_), 1.98 (s, 3H, CH_3_).

^13^C-NMR (CDCl_3_, 100 MHz): 167.3 (C=O), 136.1 [C(CH_3_)(CH_2_)], 125.9 (C=CH_2_), 70.1 (O–CH_2_),69.2 (O–CH_2_), 63.7 (O–CH_2_), 50.7 (N_3_–CH_2_), 18.3 (CH_3_).

##### 2-Methyl-acrylic acid 2-{2-[2-(2-azido-ethoxy)-ethoxy]-ethoxy}-ethyl ester **8**

Yield: 71%

^1^H-NMR (CDCl_3_, 400 MHz): 6.16 (s, 1H, CH_2_), 5.60 (s, 1H, CH_2_), 5.33 (t, *J* = 4.9 Hz, 2H, O–CH_2_), 3.77 (t, *J* = 3.8 Hz, 2H, O–CH_2_), 3.70 (s, 10H, O–CH_2_), 3,41 (t, *J* = 5.0 Hz, 2H, CH_2_–N_3_), 1.97 (s, 3H, CH_3_).

^13^C-NMR (CDCl_3_, 100 MHz): 136.2 [C(CH_3_)(CH_2_)], 125.8 (C=CH_2_), 70.8 (O–CH_2_), 70.1 (O–CH_2_), 69.2 (O–CH_2_), 63.8 (O-CH_2_), 50.7 (N_3_–CH_2_), 18.3 (CH_3_).

##### 2-Methyl-acrylic acid 2-[2-(2-{2-[2-(2-azido-ethoxy)-ethoxy]-ethoxy}-ethoxy)-ethoxy]-ethyl ester **9**

Yield: 55%

^1^H-NMR (CDCl_3_, 400 MHz): 6.17 (s, 1H, CH_2_), 5.60 (s, 1H, CH_2_), 4.34 (t, *J* = 4.7 Hz, 2H, O–CH_2_), 3.78 (t, *J* = 4.7, 2H, O–CH_2_), 3.71 (t, *J* = 5.0, 2H, O–CH_2_), 3.40 (t, *J* = 4.9, 2H, CH_2_–N_3_), 1.98 (s, 3H, CH_3_).

^13^C-NMR (CDCl_3_, 100 MHz): 167.4 (C=O), 136.2 [C(CH_3_)(CH_2_)], 125.7 (C=CH_2_), 70.7 (O–CH_2_), 70.6 (O–CH_2_), 70.0 (O–CH_2_), 69.2 (O–CH_2_), 63.9 (O–CH_2_), 50.7 (N_3_–CH_2_), 18.3 (CH_3_).

##### 2-Methyl-acrylic acid 8-(2-{2-[2-(2-azido-ethoxy)-ethoxy]-ethoxy}-ethoxy)-octyl ester **10**

Yield: 66%

^1^H-NMR (CDCl_3_, 400 MHz): 6.11 (s, 1H, CH_2_), 5.56 (s, 1H, CH_2_), 4.15 (t, *J* = 6.7 Hz, 2H, O–CH_2_), 3.72–3.64 (m, 14H, O–CH_2_), 3.62–3.57 (m, 2H, O–CH_2_), 3.46 (t, *J* = 6.8 Hz, 2H, O–CH_2_), 3.41 (t, *J* = 5.1 Hz, 2H, N_3_–CH_2_), 1.96 (s, 2H, O–CH_2_), 1.73–1.64 (m, 2H, O–CH_2_), 1.62–1.57 (m, 2H, O–CH_2_), 1.44–1.26 (m, 8H, O–CH_2_).

^13^C-NMR (CDCl_3_, 100 MHz): 167.56 (C=O), 136.55 [C(CH_3_)(CH_2_)], 125.16 (C=CH_2_), 71.48 (O–CH_2_), 70.72 (O–CH_2_), 70.70 (O–CH_2_), 70.65 (O–CH_2_), 70.64 (O–CH_2_), 70.62 (O–CH_2_), 70.08 (O–CH_2_), 70.05 (O–CH_2_), 64.80 (O–CH_2_), 50.70 (N_3_–CH_2_), 29.61 (CH_2_), 29.36 (CH_2_), 29.21 (CH_2_), 28.60 (CH_2_), 26.02 (CH_2_), 25.93 (CH_2_), 18.34 (CH_3_).

#### 2.1.6. General Procedure for Click Reaction of Azidomethacrylates with **13**

The click reaction was carried out under optimized conditions according to Schmidt et al. 2014 [62]. Azido methacrylate (1 eq) and propargyl GlcNAc **13** (1 eq) were dissolved in a mixture of dichloromethane/methanol/water (10:10:3). Then, CuSO_4_ (0.04 eq), tris(benzyltriazolylmethyl)amine (TBTA) (0.01 eq), and sodium ascorbate (0.22 eq) were added, and the mixture was heated to reflux for 1 h. After cooldown and adding water, the solution was extracted 3 times with dichloromethane, the organic layer dried over Na_2_SO_4_, and the solvent evaporated in vacuum. Column chromatography yielded the products as yellowish oil.

##### 2-Methyl-acrylic acid 2-{2-[4-(3-acetylamino-4,5-dihydroxy-6-hydroxymethyl-tetrahydro-pyran-2-yloxymethyl)-[1,2,3]triazol-1-yl]-ethoxy}-ethyl ester **11a**

Yield: 75%

^1^H-NMR (CDCl_3_, 400 MHz): 7.67 (s, 1H, Ar-H), 6.03 (s, 1H, CH_2_), 5.84 (s, 1H, H-1), 5.54 (s, 1H, CH_2_), 5.16 (t, *J* = 9.2 Hz, 1H, H-3), 5.04 (t, *J* = 9.4 Hz, 1H, H-4), 4.83–4.75 (m, 3H, O–CH_2_ + H-2), 4.49 (s, 2H, O–CH_2_), 4.24–4.19 (m, 2H, O–CH_2_), 4.07 (dd, *J* = 16.3, 9.5 Hz, 1H, H-6), 3.92 (s, 1H, H-6), 3.82 (s, 2H, O–CH_2_), 3.68–3.65 (m, 1H, H-5), 3.63 (t, *J* = 4.7 Hz, 2H, O–CH_2_), 2.03 (s, 3H, C(O)CH_3_), 1.96 (s, 3H, C(O)CH_3_), 1.95 (s, 3H, C(O)CH_3_), 1.87 (s, 3H, C(O)CH_3_), 1.79 (s, 3H, CH_3_).

##### 2-Methyl-acrylic acid 2-[2-(2-{2-[4-(4,5-diacetoxy-6-acetoxymethyl-3-acetylamino-tetrahydro-pyran-2-yloxymethyl)-[1,2,3]triazol-1-yl]-ethoxy}-ethoxy)-ethoxy]-ethyl ester **11b**

Yield: 63%

^1^H-NMR (CDCl_3_, 400 MHz): 7.58 (s, 1H, Ar-H), 5.96 (s, 1H, CH_2_), 5.71 (d, *J* = 8.5 Hz, 1H, H-1), 5.42 (s, 1H, CH_2_), 5.05 (t, *J* = 9.9 Hz, 1H, H-3), 4.95 (t, *J* = 9.6 Hz, 1H, H-4), 4.78 (d, *J* = 12.3 Hz, 1H, H-2), 4.68 (dd, *J* = 23.6, 10.5 Hz, 2H, O–CH_2_–Ar), 4.39 (t, *J* = 4.7 Hz, 2H, O–CH_2_), 4.17–4.10 (m, 3H, O–CH_2_ + H-6), 3.99 (dd, *J* = 12.4, 2.1 Hz, 1H, H-6), 3.82 (dd, *J* = 18.0, 8.5 Hz, 1H, H-5), 3.72 (t, *J* = 5.0 Hz, 2H, O–CH_2_), 3.60–3.57 (m, 2H, O–CH_2_), 3.53–3.45 (m, 10H, O–CH_2_), 1.94 [s, 2H, C(O)CH_3_], 1.87 [s, 3H, C(O)CH_3_], 1.86 [s, 3H, C(O)CH_3_], 1.79 [s, 3H, C(O)CH_3_], 1.69 (s, 3H, CH_3_).

##### 2-Methyl-acrylic acid 2-(2-{2-[2-(2-{2-[4-(3-acetylamino-4,5-dihydroxy-6-hydroxymethyl-tetrahydro-pyran-2-yloxymethyl)-[1,2,3]triazol-1-yl]-ethoxy}-ethoxy)-ethoxy]-ethoxy}-ethoxy)-ethyl ester **11c**

Yield: 56%

^1^H-NMR (CDCl_3_, 400 MHz): 7.76 (s, 1H, Ar–H), 6.12 (s, 1H, CH_2_), 6.05 (d, *J* = 8.4 Hz, 1H, H-1), 5.57 (d, *J* = 1.6 Hz, 1H, CH_2_), 5.23 (t, *J* = 9.9 Hz, 1H, H-3), 5.09 (t, *J* = 9.6 Hz, 1H, H-4), 4.94 (d, *J* = 12.6 Hz, 1H, H-2), 4.84 (dd, *J* = 33.4, 10.4 Hz, 2H, O–CH_2_–Ar), 4.54 (t, *J* = 4.8 Hz, 2H, O–CH_2_), 4.32–4.23 (m, 3H, O–CH_2_ + H-6), 4.14 (dd, *J* = 12.4, 2.1 Hz, 1H, H-6), 3.87 (t, *J* = 5.0 Hz, 2H, O–CH_2_), 3.77–3.71 (m, 3H, O-CH_2_ + H-5), 3.68–3.59 (m, 20H, O–CH_2_), 2.09 (s, 3H, C(O)CH_3_), 2.02 (s, 3H, C(O)CH_3_), 2.01 (s, 3H, C(O)CH_3_), 1.94 (s, 3H, C(O)CH_3_), 1.85 (s, 3H, CH_3_).

##### 2-Methyl-acrylic acid 8-{2-[2-(2-{2-[4-(4,5-diacetoxy-6-acetoxymethyl-3-acetylamino-tetrahydro-pyran-2-yloxymethyl)-[1,2,3]triazol-1-yl]-ethoxy}-ethoxy)-ethoxy]-ethoxy}-octyl ester **11d**

Yield: 86%

^1^H-NMR (CDCl_3_, 400 MHz): 7.61 (s, 1H, Ar-H), 5.94 (s, 1H, CH_2_), 5.85 (d, *J* = 8.6 Hz, 1H, H1), 5.39 (s, 1H, CH_2_), 5.08 (t, *J* = 9.9 Hz, 1H, H3), 4.94 (t, *J* = 9.6 Hz, 1H, H4), 4.82–4.60 (m, 3H, H2 + Ar–CH_2_–O), 4.41–4.34 (m, 2H, O–CH_2_), 4.25–4.08 (m, 2H, H6), 4.02–3.92 (m, 2H, O–CH_2_), 3.63–3.54 (m, 1H, H5), 3.51–3.45 (m, 10H, O–CH_2_), 3.43–3.39 (m, 2H, O–CH_2_), 3.28 (t, *J* = 6.8 Hz, 2H, O–CH_2_), 1.94 (s, 3H, CH_3_), 1.87 (s, 3H, CH_3_), 1.86 (s, 3H, CH_3_), 1.78 (s, 3H, CH_3_), 1.69 (s, 3H, CH_3_), 1.56–1.37 (m, 5H, CH_2_), 1.22–1.13 (m, 9H, CH_2_).

#### 2.1.7. General Procedure for Click Reaction of Azidomethacrylates with Propargyl Alcohol

The click reaction with propargyl alcohol was carried out under conditions as previously published [62]. Briefly, azido methacrylate (1 eq) and propargyl alcohol (1.3 eq) were dissolved in a mixture of dichloromethane/methanol/water (10:10:3). Then, CuSO_4_ (0.04 eq), TBTA (0.01 eq), and sodium ascorbate (0.22 eq) were added, and the mixture was heated to reflux overnight. After cooldown and adding water, the solution was extracted 3 times with dichloromethane, the organic layer dried over Na_2_SO_4_, and the solvent evaporated in vacuo. Column chromatography (ethyl acetate/methanol, 4:1) yielded the products as yellow oils.

##### 2-Methyl-acrylic acid 2-[2-(4-hydroxymethyl-[1,2,3]triazol-1-yl)-ethoxy]-ethyl ester **12a**

Yield: 18%

^1^H-NMR (CDCl_3_, 200 MHz): 7.68 (s, 1H, Ar–H), 6.10 (dd, *J* = 1.5, 1.0 Hz, 1H, CH_2_), 5.60 (p, *J* = 1.6 Hz, 1H, CH_2_), 4.77 (s, 2H, CH_2_), 4.54 (t, *J* = 5.0 Hz, 2H, O–CH_2_), 4.29 (t, *J* = 4.7 Hz, 2H, O–CH_2_), 3.87 (t, *J* = 5.1 Hz, 2H, O–CH_2_), 3.69 (ddd, *J* = 5.4, 4.0, 2.1 Hz, 2H, O–CH_2_), 1.94 (dd, *J* = 1.5, 1.0 Hz, 3H, CH_3_).

^13^C-NMR (CDCl_3_, 50 MHz): 125.88 (C=CH_2_), 115.58, 69.40 (O–CH_2_), 69.20 (O–CH_2_), 69.04 (O–CH_2_), 63.42 (O–CH_2_), 50.30 (N–CH_2_), 18.21 (CH_3_).

##### 2-Methyl-acrylic acid 2-[2-(4-hydroxymethyl-[1,2,3]triazol-1-yl)-ethoxy]-ethyl ester **12b**

Yield: 39%

^1^H-NMR (CDCl_3_, 200 MHz): 7.76 (s, 1H, Ar–H), 6.11 (dd, *J* = 1.5, 0.9 Hz, 1H, CH_2_), 5.57 (p, *J* = 1.6 Hz, 1H, CH_2_), 4.78 (s, 2H, CH_2_), 4.53 (t, *J* = 5.3 Hz, 2H, CH_2_), 4.29 (dd, *J* = 5.7, 4.2 Hz, 2H, CH_2_), 3.86 (t, *J* = 5.2 Hz, 2H, CH_2_), 3.74 (dd, *J* = 5.5, 4.2 Hz, 2H, CH_2_), 3.67–3.57 (m, 8H, CH_2_), 1.93 (s, 3H, CH_3_).

^13^C-NMR (CDCl_3_, 50 MHz): 125.68 [C(CH_3_)(CH_2_)], 122.87 (C=CH_2_), 70.62 (O–CH_2_), 70.58 (O–CH_2_), 70.51 (O–CH_2_), 70.48 (O–CH_2_), 69.41 (O–CH_2_), 69.12 (O–CH_2_), 63.74 (O–CH_2_), 56.52 (O–CH_2_), 50.23 (N–CH_2_), 18.21 (CH_3_).

##### 2-Methyl-acrylic acid 2-{2-[2-(2-{2-[2-(4-hydroxymethyl-[1,2,3]triazol-1-yl)-ethoxy]-ethoxy}-ethoxy)-ethoxy]-ethoxy}-ethyl ester **12c**

Yield: 96%

^1^H-NMR (CDCl_3_, 600 MHz): 7.86 (s, 1H, Ar–H), 6.12 (dd, *J* = 1.6, 1.0 Hz, 2H, CH_2_), 5.56 (q, *J* = 1.6 Hz, 2H, CH_2_), 4.79 (s, 2H, CH_2_), 4.54 (t, *J* = 4.9 Hz, 2H, CH_2_), 4.30–4.27 (m, 2H, CH_2_), 3.86 (t, *J* = 4.9 Hz, 2H, CH_2_), 3.72 (dd, *J* = 5.5, 4.3 Hz, 2H, CH_2_), 3.65–3.59 (m, 16H, CH_2_), 2.37 (s, 1H, OH), 1.94 (dd, *J* = 1.5, 1.0 Hz, 3H, CH_3_).

^13^C-NMR (CDCl_3_, 200 MHz): 136.12 [C(CH_3_)(CH_2_)], 125.72 (C=CH_2_), 70.60 (O–CH_2_), 70.57 (O–CH_2_), 70.56 (O–CH_2_), 70.54 (O–CH_2_), 70.53 (O–CH_2_), 70.48 (O–CH_2_), 70.46 (O–CH_2_), 70.38 (O–CH_2_), 69.38 (O–CH_2_), 69.10 (O–CH_2_), 64.34 (O–CH_2_), 63.81 (O–CH_2_), 57.20 (O–CH_2_), 56.62 (O–CH_2_), 50.34 (N_3_–CH_2_), 30.57 (CH_2_), 18.28 (CH_3_).

#### 2.1.8. General Procedure for Free Radical Polymerization

Combined monomers (in general, dimethacrylamide, benzophenone methacrylate **14** and functional methacrylate **11a**–**d** or **12a**–**c** in given ratios (Table 1) were dissolved in water-free THF under Ar atmosphere to a total monomer concentration of 2 M. Azobisisobutyronitrile (AIBN) (0.01 mol%) was added and the reaction mixture heated to reflux for 16 h. Cooldown followed by precipitation of the polymers in 10-fold excess iso-hexane yielded the product as a white precipitate. The precipitate was dissolved in water and lyophilized to obtain the product as a white powder.

#### 2.1.9. General Deprotection Procedure of GlcNAc Polymers

Deprotection was carried out according to a standard Zemplén procedure [63]. GlcNAc polymers were dissolved in dry methanol in a sealed tube under Ar atmosphere. NaOMe (30% solution in methanol; 0.2 eq referring to glycoside content) was added and the mixture stirred at room temperature overnight. Water was added until the precipitated polymers were dissolved. Addition of ion exchange resin (Dowex 50WX8, 200–400 mesh, Carl Roth, Karlsruhe, Germany) followed by filtration and lyophilization yielded the products as yellowish powders. The crude polymer was further purified by 3 times dissolving in methanol and precipitation in 10-fold excess of Et_2_O, followed by dissolution in ddH_2_O and lyophilization. Pure polymers were obtained as white powder.

### 2.2. Preparation of Polymer Coatings

Polymers were diluted to a concentration of 25 or 5 mg/mL in a H_2_O/ethanol 5:1 mixture. The mixture was sterile filtered before use. From the mixture, 20 µL was pipetted into each well of a 48-well plate, 34.6 µL into each well of a 24-well plate, or 5.76 µL into each well of a 96-well plate. The plates were allowed to dry under sterile conditions for at least 4 h and crosslinked with 3 J/cm^2^ UV light at 254 nm, followed by 3× washings with 250 µL of phosphate-buffered saline (PBS).

Coverslips were coated by carefully pipetting 10 µL of each polymer solution on a 13-mm PETG coverslip (Tissue Culture Coverslips 13 mm, Sarstedt, Nümbrecht, Germany) to obtain a fully coated surface. The coverslips were let dry in air for at least 4 h, followed by crosslinking with 3 J/cm^2^ at 254 nm. Washing 3× with ddH_2_O and 3× with ethanol, followed by drying in an N_2_ stream yielded the final coating, which was used directly for IR spectroscopy.

### 2.3. Physicochemical Surface Characterization

IR data was recorded on a Tensor 27 FT-IR Spectrometer (Bruker, Germany). Scanning electron microscope (SEM) images were obtained with an XL-30 SEM (Philips, Amsterdam, Netherlands) at 10 kV. The samples were dried in vacuum and thereafter coated with an approx. 5 nm thick Au/Pd layer (SC7620 sputter coater, Quorum, Laughton, UK). Images were taken at a 40° tilted angle. Atomic force microscope (AFM) images were obtained using a CoreAFM (Nanosurf, Liestal, Switzerland) with a TAP150GD-G tip (BudgetSensors, Sofia, Bulgaria, tip radius <10 nm) in tapping mode.

### 2.4. Biological Evaluation

#### 2.4.1. L-929 Mouse Fibroblast Cell Culture

L-929 mouse fibroblasts were a gift from Dr. Oliver Podlech (CleanControlling GmbH, Emmingen-Liptingen, Germany). Media and reagents were purchased from Sigma-Aldrich (Taufkirchen, Germany). Sterile cell cultureware was purchased from VWR, Germany. Fibroblasts were cultured in low-glucose Dulbecco’s Modified Eagle Medium (DMEM), supplemented with 10% (*v*/*v*) fetal calf serum (FCS), 1% (*v*/*v*) penicillin-streptomycin (10,000 U/mL) and 1% (*v*/*v*) l-glutamine. Cells were incubated at 37 °C and 5% CO_2_ in a humidified incubator (CB series C150, Binder, Tuttlingen, Germany) and subculturing was performed using trypsin/Ethylenediaminetetraacetic acid (EDTA) before reaching confluency, approximately every third day.

#### 2.4.2. Extract Test Using the MTT Assay

Polymer extracts were prepared according to USP standard [64]. Briefly, after coating a 24-well plate with polymers followed by washing steps, 317 µL of DMEM (20 mL for 120-cm^2^ coated surface) was added and the coating incubated for 24 h at 37 °C in a humidified 5% CO_2_ atmosphere. A cell suspension of L-929 in DMEM (100,000 cells/mL) was added to an uncoated 96-well plate (treated for cell culture, 100 µL/well) and grown to adherence overnight. Medium in each well was replaced by prepared extract medium (100 µL) or medium containing 6% Dimethyl sulfoxide (DMSO) for the cytotoxicity positive control and incubated for 72 h at 37 °C in a humidified 5% CO_2_ atmosphere. After 72 h, medium was replaced by 110 µL of DMEM containing 10% of a 10mM MTT solution in PBS. After incubating for 4 h in the incubator, 100 µL of 10% SDS in 0.01M HCl solution was added and incubated for 4 h. Absorbance was measured at 570 nm using a Tecan Infinite M2000 microplate reader. Cell viability was calculated as the percentage ratio of averaged absorbance of triplicate wells containing extract versus the averaged absorbance of untreated control wells.

#### 2.4.3. Direct Contact Test Using the MTT Assay

Cell suspension (100,000 cells/mL) was added to a polymer-coated 96-well plate (treated for cell culture, 100 µL each) and incubated over 24 and 48 h at 37 °C and 5% CO_2_ in a humidified incubator. At the end of incubation, the medium was removed, and 110 µL of medium containing 10% of a 10 mM MTT solution in PBS was added. After incubating the cells for 4 h at 37 °C in a humidified 5% CO_2_ atmosphere, 100 µL of 10% SDS in 0.01M HCl was added and incubated for 4 h at 37 °C in 5% CO_2_. The absorbance was measured at 570 nm using a microplate reader. Cell viability was calculated as the percentage ratio of averaged absorbance of each triplicate well containing the same polymer coating versus the averaged absorbance of uncoated control wells.

#### 2.4.4. Bacterial Cell Culture

For antimicrobial tests, bacteria cell lines of *Staphylococcus aureus* (MRSA, DSM 28766) and *Escherichia coli* (K12, DSM 498) were used. Bacterial strains were stored at −80 °C in glycerol stocks. For each experiment, a new vial of bacterial strain was thawed and incubated (Minitron, Infors HT, Bottmingen, Switzerland) overnight at 37 °C and 100 rpm in LB medium before use in the experiments.

#### 2.4.5. Antibacterial Assay by Optical Density

The antibacterial effects of the polymers were evaluated using a direct contact method according to ISO 22196 [65] with a thin film of bacteria solution in LB medium (high-nutrition) or PBS (low-nutrition) between the polymer to be analyzed and a polymer slide to ensure direct contact. A 24-well cell culture tissue plate coated with the polymers to be tested was inoculated with 100 µL of bacterial suspension at a concentration of 3 × 10^5^ cells/mL and sealed with a PETG coverslip. Bacterial solutions were prepared in LB medium for the high-nutrition condition and in PBS for the low-nutrition condition. As controls, wells without polymer coating were treated with bacteria suspension and medium without cells. After incubating the plate for 24 h at 37 °C and 90% humidity, bacteria were removed from the plates by addition of 1 mL of soybean casein digest lecithin polysorbate broth (SCDLP), followed by pipetting up and down 4 times to detach all bacteria. From this mixture, 200 µL was transferred to a 96-well plate in a series of dilutions. The 96-well plate was sealed with parafilm and placed in a plate reader preheated to 37 °C. Optical density at 600 nm was measured every 30 min over the next 12 h. The plate was shaken briefly every 10 min to ensure distribution of nutrients. Measured values from each sample were compared to determine the viability relative to that of untreated samples. The evaluation time point was chosen to be in the exponential phase before reaching the inflection point. For evaluation, the last time point was used for which Equation (1) was still fulfilled:

(1) $$\frac{\log\left({\mathrm{OD}}_{600\mathrm{nm},t_{+1}}\right)-2\times \log\left({\mathrm{OD}}_{600\mathrm{nm},t}\right)+\log\left({\mathrm{OD}}_{600\mathrm{nm},t_{-1}}\right)}{t-t_{-1}}>0$$

where OD_600nm_ is the optical density at 600 nm for the different measuring points; t is the measuring time of the data point to be evaluated; t_+1_ is the measuring point of the subsequent data point and t_−1_ is the measuring point of the previous data point.

#### 2.4.6. Antibacterial Assay by Colony-Forming Units

The antibacterial effects of the polymers were evaluated using a direct contact method as a droplet of bacteria solution in PBS on top of the polymer. A bacterial overnight culture in LB medium was centrifuged (10 min, 4000× *g*) and resuspended in PBS to an OD_600_ value of 0.2. Coated and uncoated PETG coverslips were inoculated in a 6-well tissue culture plate with 100 µL of the prepared bacteria suspension. Uncoated PETG coverslips were used as reference. The samples were cultured for 24 h at 37 °C and 90% humidity in a humid chamber. Solutions were removed and transferred to a sterile tube. Each coverslip was transferred to a 15 mL Falcon tube, covered with 900 µL of PBS, and treated in an ultrasonic bath at 50 Hz for 15 min to remove bacteria. Both PBS fractions were combined, vortexed for 1 min, and pipetted in a series of dilutions on LB agar plates in duplicate (100 µL per dilution and plate). Agar plates were cultured at 37 °C in an incubator, followed by counting of colony-forming units (CFU) after 24 h.

#### 2.4.7. Crystal Violet Assay for Biofilm Assessment

Biofilm formation was assessed in 96-well plates by staining with crystal violet dye. Briefly, 200 µL of a bacterial overnight culture, adjusted to a concentration of 3 × 10^5^ cells/mL in lysogeny broth (LB) medium, was added to each sample. Empty wells (in the outer row, in particular) were filled with 200 µL of PBS to prevent the samples from drying out. The closed well plate was incubated for 24, 48, or 72 h at 37 °C in an incubator without shaking. Then, OD_600_ was measured to ensure comparable cell growth in each well. The medium was gently discarded without removing the biofilm, and the samples were washed carefully 3 times with PBS, followed by fixing with 200 µL of absolute ethanol. The ethanol was aspirated, and the samples were dried for 10 min under sterile conditions. For biofilm staining, 200 µL of 0.5 wt% (*wt*/*vol*) crystal violet staining solution in PBS was added to each sample, and the plate was incubated for 2 min at room temperature. The staining solution was removed and the samples washed 6× with 200 µL of PBS to remove excess dye. The samples were left to dry overnight under a sterile bench, followed by addition of 100 µL of ethanol to release the dye. After a 10 min incubation, the mixture in each well was transferred to a new 96-well plate, and absorbance at 595 nm was measured using a plate reader.

#### 2.4.8. Extracellular Polymeric Substance (EPS) Assessment by Phenol-Sulfuric Acid Method

In addition, the biofilm formation was assessed by analyzing the carbohydrates in the formed biofilm. Therefore, the phenol-sulfuric acid method according to Masuko et al. [66] was performed. Briefly, polymer coatings were treated in a 96-well plate as previously described for the crystal violet assay and incubated for 24, 48 and 72 h at 37 °C in an incubator without shaking. OD_600_ was measured to ensure comparable cell growth in each well, and the medium was gently discarded. After 3 wash steps with sterile PBS, the samples were fixed with 200 µl of absolute ethanol. The ethanol was gently aspirated, and the samples were dried for 10 min under sterile conditions. A volume of 150 µl of concentrated sulfuric acid was added to each well, immediately followed by 30 µl of 5% phenol in water. The plate was incubated at 90 °C for 5 min and then cooled in an ice bath for an additional 5 min. The absorbance at 490 nm was measured using a plate reader to quantify EPS.

#### 2.4.9. Live/Dead Staining

Besides the Crystal violet staining and EPS assessment, we also performed live/dead staining using the bacteria live/dead staining kit (PromoCell GmbH, Heidelberg, Germany). Polymer coatings were treated in a 96-well plate as previously described for the crystal violet assay and incubated for 24, 48 and 72 h at 37 °C in an incubator without shaking and stained accordingly to the manufacturer’s instructions. Briefly, biofilm samples were washed 3 times with sterile 150 mM NaCl solution and stained for 15 min at room temperature in the dark with an appropriate mixture of DMAO (ex/em 490/540) and EthD-III (ex/em 530/630). Live bacteria with an intact cell membrane are stained fluorescent green, whereas dead bacteria with a disrupted cell membrane are stained fluorescent red. Labeled cells were imaged using the fluorescent microscope Observer.Z1 (Carl Zeiss AG, Oberkochen, Germany) and processed using the software ZEN blue edition (Version 3.4, Carl Zeiss AG, Oberkochen, Germany).

#### 2.4.10. Statistical Analysis

Measurements for biological evaluation (bacterial and cell culture) were replicated with *n* = 3 and expressed as mean ± standard deviation (SD) unless stated otherwise. Statistical significance was analyzed with pairwise Student’s *t*-test, and statistically significant values were defined as *p* < 0.05 (*).

## 3. Results and Discussion

### 3.1. Monomer Synthesis

**Figure 1:**
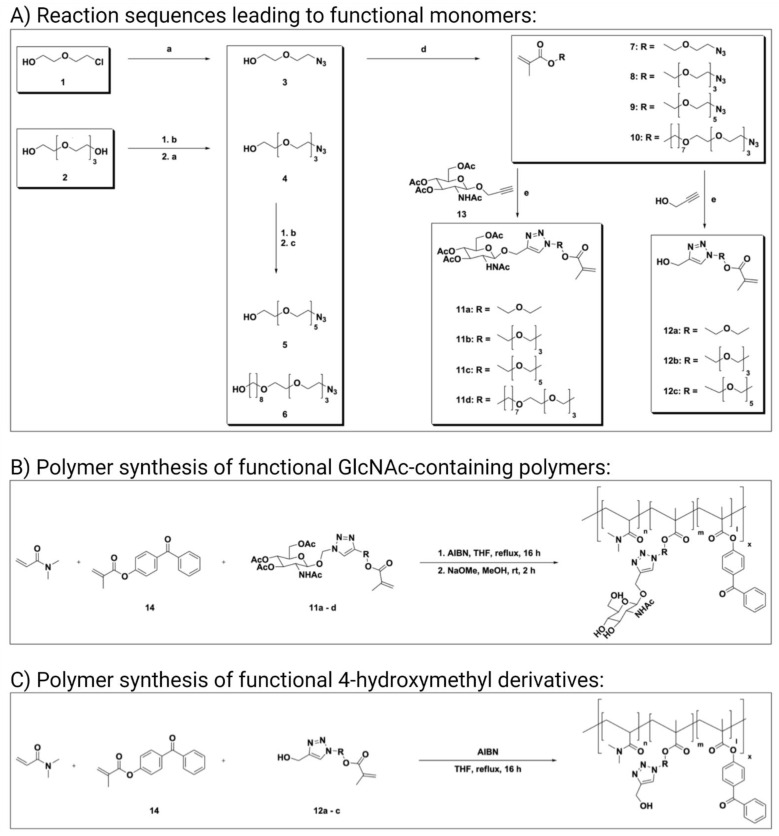
Chemical Synthesis. (**A**) Reaction sequences leading to functional monomers **11a**–**d** or **12a**–**c**, respectively. Reaction conditions: (**a**) NaN_3_, CH_3_CN, reflux, 16 h; (**b**) TsCl, NEt_3_, CH_2_Cl_2_, rt, 16 h; (**c**) NaH, THF, reflux, 48 h; (**d**) methacryloyl chloride, NEt_3_, CH_2_Cl_2_, 0 °C–rt, 16 h; and (**e**) CuSO_4_, TBTA, Na ascorbate, H_2_O/MeOH/CH_2_Cl_2_ 3:10:10, 60 °C, 1 h. (**B**) Polymer synthesis of functional *N*-acetylglucosamine (GlcNAc)-containing polymers. Benzophenone methacrylate **14** was copolymerized with glycosidic monomers **11a**–**d** by free radical polymerization with AIBN, followed by Zemplén deprotection. (**C**) Polymer synthesis of functional 4-hydroxymethyl derivatives using 4-hydroxymethyl-[1,2,3]-triazo-1-yl derivatives of functional monomers **12a**–**c** of the polymers with AIBN.

Azido linkers **3**–**6** were synthesized by sequential tosylation steps, followed by substitution with either sodium azide or another linker fragment. Those prolonged linkers were reacted to azido methacrylate **7**–**10**, followed by click reaction to either functional GlcNAc methacrylate **11a**–**d** or their corresponding 4-hydroxymethyl methacrylate derivatives **12a**–**c**. The detailed reaction sequences are shown in Figure 1.

Azido linker **3** was synthesized by reacting 2-(2-chloroethoxy)-ethanol with sodium azide (**a**) according to the literature [60]. Azido linkers **4**–**6** were synthesized via sequential tosylation (**b**) and substitution steps with either sodium azide (**a**) or another linker for chain prolongment. For the azido hexaethylene glycol linker **5**, diethylene glycol was used for chain prolongment as published Mahou et al. [59], and for the difunctional azido tetraethyleneglycol octyl linker **6**, 1,8-octanediol was used for chain prolongment. Azidomethacrylates **7**–**10** were synthesized following published protocols, using methacryloyl chloride and triethylamine [61].

The functional glycoside, propargyl GlcNAc **13**, was synthesized as described by Schmidt et al. [62]. *N*-Acetylglucosamine was used as starting material, followed by protection with acetyl groups, conversion of the peracetylated *N*-acetylglucosamine into an oxazoline as glycoside donor, and further glycosidation using propargyl alcohol to *N*-acetylpropargylglucosamine **13**. For click functionalization, the optimized conditions reported by Schmidt et al. [62] were used, yielding the GlcNAc-functionalized monomers **11a**–**d**. To further elucidate antimicrobial properties of the combined triazole and linker in the polymers, azido oligoethylene glycol methacrylates **7**–**9** were reacted under the same conditions with propargyl alcohol to their 4-hydroxymethyl-[1,2,3]-triazo-1-yl counterparts **12a**–**c**. The 4-hydroxymethyl derivatives **12a**–**c** and the GlcNAc derivatives **11a**–**d** were used as functional monomers directly for polymerization.

### 3.2. Polymer Synthesis

Functional GlcNAc monomers **11a**–**d** were successfully polymerized using free radical polymerization with AIBN as radical starter (Figure 1B), followed by several purification steps with precipitation and *O*-acetyl deprotection to the functional glycosidic polymer. Functional 4-hydroxymethyl monomers **12a**–**c** were polymerized using free radical polymerization with AIBN (Figure 1C) followed by purification and used directly because no protection groups were involved. The 4-methacryloyloxy-benzophenone **14** was synthesized according to the literature [57]. All synthesized polymers with their corresponding abbreviations are listed in Table 1.

**Figure 2:**
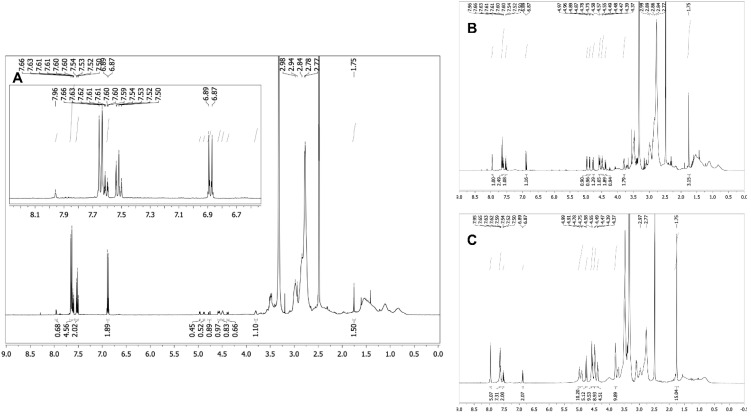
1H-NMR spectra (600 MHz) of the 5%—(**A**), 10%—(**B**) and 25%—(**C**) GlcNAc-4EG polymers (Table 1). The GlcNAc and 4-hydroxymethyl triazoles proton gave a distinct singlet at 7.96 ppm, whereas the benzophenones aromatic protons gave signals from 7.66 to 6.67 ppm. Furthermore, the GlcNAc acetyl group singlet showed a signal at 1.75 ppm. The broad multiplet from 2.98 to 2.75 ppm belongs to the dimethacrylamide methyl groups. Copolymer ratio was calculated by the integral ratios of triazole-H vs. NAc vs. aromatic benzophenone-H. For the 4-hydroxymethyl comonomers, only triazole-H was integrated vs. aromatic benzophenone-H. The figure shows the increasing triazole singlet (7.96 ppm) and NAc singlet (1.75 ppm) with increasing comonomer ratio of **11b** vs. the aromatic protons of MBP **14**. The dimethacrylamide multiplet between 2.98 and 2.75 ppm was relatively constant in all three spectra because it overlays the tetraethylene glycol.

Analysis of the polymers and the copolymer ratio between MBP **14** and functional monomers **11** or **12** was performed by ^1^H NMR spectroscopy, followed by 2-dimensional measurements for glycosidic structure determination. The triazole proton, showing a relatively isolated singlet at 7.96 ppm, was integrated against the benzophenone aromatic protons and against the *N*-acetyl group of the glycosidic monomers **11a–d**. For the 4-hydroxymethyl derivatives **12a–c** without glycoside, the triazole proton at 7.96 ppm was integrated against the benzophenone protons only. The dimethacrylamide methyl (DMAm) groups showed a broad multiplet at 2.98–2.75 ppm, which overlapped with the ethylene glycol signals of comonomers **11** and **12**; therefore, the integral ratio of those signals did not match the actual copolymer ratio as shown in Figure 2. The multiplet integral was relatively constant over all three copolymers of **11b**, which had decreasing dimethacrylamide content from 90% to 70%, whereas the content of **11b** with a tetraethylene glycol linker increased from 2.5% to 25%. Therefore, the DMAm content was not calculated using the integral ratios. Successful deprotection of GlcNAc-containing polymers was confirmed by disappearance of the O-acetyl groups in ^1^H NMR after the deprotection step. For the 4-hydroxymethyl-derivative comonomers **12a–c**, adjusted copolymer ratios of 1:1 MBP **14** vs. **12** were obtained. In the GlcNAc copolymer group, consisting of copolymers with comonomers **11a–d**, different copolymer ratios of MBP **14** vs. **11** were obtained. Possible mechanisms are ester hydrolysis during the Zemplén deprotection step of the GlcNAc residue, because the 4-hydroxymethyl–containing polymeric counterparts did not show different copolymer ratios of the benzophenone. Interestingly, the GlcNAc-4EG copolymers showed no reduction in MBP content relative to the GlcNAc residue (Figure 2). Therefore, different reactivities of benzophenone in combination with several GlcNAc-methacrylates and oligoethylene glycol linkers are possible explanations.

Interestingly, the copolymerization of 50% GlcNAc-4EG-methacrylate monomer resulted in ester hydrolysis of the methacrylate ester during polymerization, leading to the propargyl GlcNAc tetraethylene glycol clickamer **15** (data not shown, cf. Supplementary Materials), following the same workup procedure as for the other polymers.

### 3.3. Coating of PETG Coverslips

**Figure 3:**
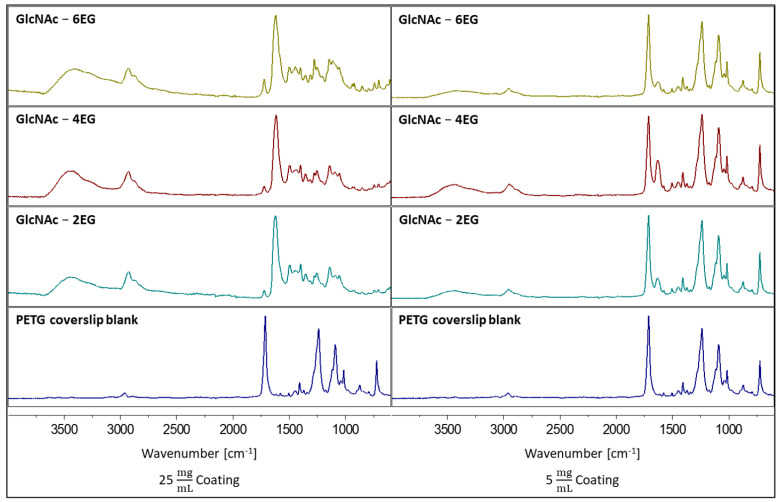
Recorded Fourier transform-infrared spectra of selected functional PDMAm-co-P-benzophenone-MA-co-P-GlcNAc-OEG-MA coatings (Table 1) containing 5% GlcNAc copolymer and linkers with 2, 4 and 6 ethylene glycol (EG) units. Coatings obtained with 25 mg mL^−1^ coatings could be analyzed properly, whereas with 5 mg mL^−1^ coatings, the terephthalate group of the PETG was the dominant signal in the recorded IR spectra.

As the model material, polyethylene terephthalate glycol (PETG) coverslips were coated with the functional polymers. Previous studies with MBP UV crosslinker in different acrylamide scaffolds indicated that 3 J/cm^2^ was the optimum dose of UV irradiation to obtain proper coating stability with minimum unreacted MBP left and minimum coating degradation [57]. Following the crosslinking protocol with UV light (3 J/cm^2^, 254 nm) and several washing steps with ddH_2_O and ethanol, stable polymer coatings were obtained using the polymers listed in Table 1. Two concentrations of polymer solutions were applied to the coverslips to obtain coatings of different thicknesses, which were investigated via IR spectroscopy. Recorded spectra of four selected coatings are shown in Figure 3.

The dominant peak at 1713 cm^−1^ of the terephthalate of PETG from the coverslip blank disappeared in all polymeric coatings obtained by drop casting a 25 mg/mL solution after crosslinking, wherein the peak of the dimethacrylamide dimethyl-carboxamide group at 1621 cm^−1^ became visible as well as the copolymer ester groups at 1721 cm^−1^. The presence of the GlcNAc residue was confirmed through the presence of glycosidic OH groups, showing broad peaks at 3450 cm^−1^ and at 2925 cm^−1^. The stable coatings were obtained using the polymers as listed in Table 1 with the given MBP copolymer ratios. Therefore, apart from the synthesis, stable coatings using a 25 mg/mL casting solution were obtained.

In coatings obtained by casting a 5 mg/mL polymer solution, the most dominant peak in the IR spectra of the coatings was the PETG terephthalate peak at 1713 cm^−1^, followed by a smaller peak of the dimethacrylamide dimethyl-carboxamide group at 1621 cm^−1^. The copolymer ester groups at 1721 cm^−1^ were not visible at all compared with those of the thicker 25 mg/mL coatings, possibly being overlaid by the dominant terephthalate peak. As a result, the polymer coatings for antimicrobial studies and cytotoxicity evaluations were prepared by casting a 25 mg/mL solution to obtain an appropriate coating thickness.

### 3.4. Surface Morphology

**Figure 4:**
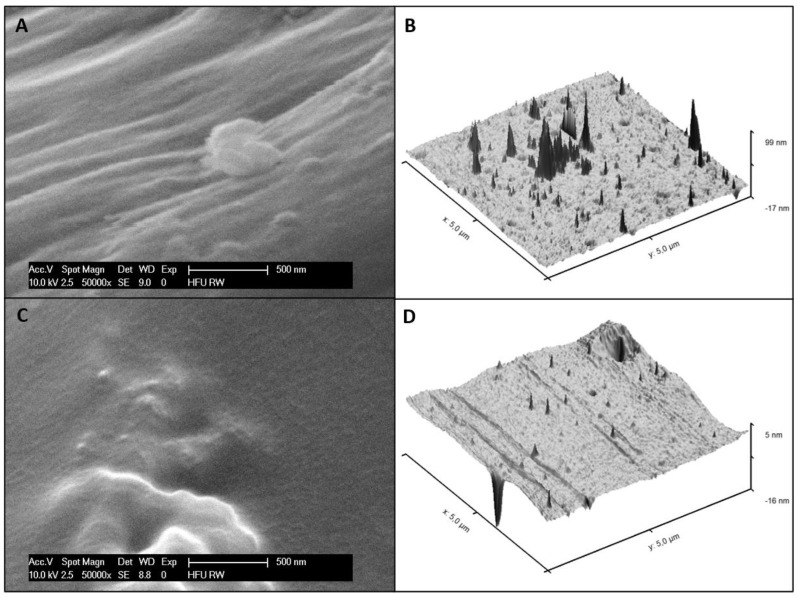
SEM (**A,C**) and AFM (**B,D**) images of the bioactive PDMAm coatings, 5%-GlcNAc-4EG (**A,B**) and GlcNAc-6EG (**C,D**).

The surface morphology of the bioactive polymer coatings 5%-GlcNAc-6EG-PDMAm and 5%-GlcNAc-4EG-PDMAm was investigated by SEM and AFM as shown in Figure 4. SEM images were recorded at a tilted angle of 40 degrees. For the 5%-GlcNAc-4EG-PDMAm, in the SEM image (Figure 4A), a textured surface showing small pores and a sponge-like structure can be observed. Furthermore, small particles in the size of up to 500 nm are present. The AFM surface topography (Figure 4B) confirms the topography. Similar observations can be made for the GlcNAc-6EG coating (Figure 4C,D), but showing less pore-like structures than the 5%-GlcNAc-PDMAm coating.

### 3.5. Coating Sterilization

Due to the sterile demands for biocompatibility testing as well as for antimicrobial activity testing against specified bacterial strains, the successful sterilization of the coating was established prior to testing. Therefore, polymer solutions were sterile filtered with a 0.2 µm sterile filter and handled under sterile condition in a biosafety cabinet during coating of the corresponding surfaces, followed by crosslinking with 3 J/cm^2^ UV-C light (254 nm). The polymer coated surfaces were then incubated for 24 h at 37 °C and further 24 h at room temperature in LB media in order to assess the sterility by absence of bacterial growth. No bacterial growth could be observed for the coated chips under these conditions.

UV sterilization is an established method in food packing, water treatment, and surface sterilization in medical settings [67,68]. For example, according to Bak et. al. [69], a 4-log fold reduction in *P. aeruginosa* in catheter disinfection was obtained, using UV-C light with a dosage of 40 mJ/cm^2^. In addition, clinical studies have shown the efficacy of UV-C light against different fungi, by using a dosage of 41.25 mJ/cm^2^ from a commercially available disinfecting device for medical settings [70,71]. In general, for a 90% inactivation of bacterial pathogens, a UV-C dosage of 8 mJ/cm^2^ is needed [71], whereas, for ssRNA viruses, an irradiation dose of 1.32–3.20 mJ/cm^2^ is needed [72]. Therefore, it can be concluded that residing pathogens as well as possible viral contaminations are eliminated after the combination of sterile filtration and UV crosslinking of the benzophenone residue in order to form the stable coating.

### 3.6. Antibacterial Activity

The antibacterial properties of the synthesized polymers were evaluated using *Escherichia coli* (*E. coli*) as a Gram-negative model organism and multidrug-resistant *Staphylococcus aureus* (MRSA) as a Gram-positive model organism. These organisms were chosen as model organisms with clinical relevance for infections and biofilm formation [73]. The bacteria were cultured in high-nutrition (LB medium) and low-nutrition (PBS) environments to assess the effect of the polymers in different nutritional conditions.

**Figure 5:**
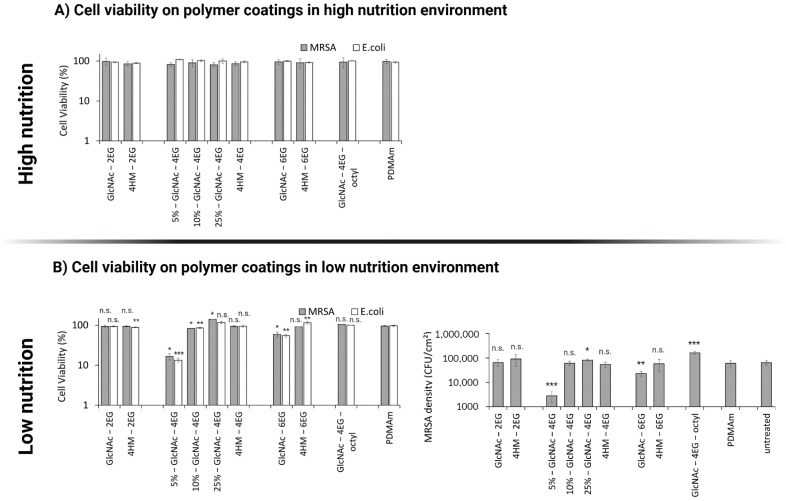
Antibacterial evaluation of polymer coatings in high- and low-nutrition environments. PDMAm corresponds to the unmodified acrylate coating. (**A**) Cell viability of *Escherichia coli* (*E. coli*) and multidrug-resistant *Staphylococcus aureus* (MRSA) on polymer coatings in the high-nutrition environment was assessed according to antibacterial assay (optical density, OD). Values are shown relative to that of the untreated sample. (**B**) Cell viability on polymer coatings in the low-nutrition environment was assessed for E. coli (*n* = 4) and MRSA (*n* = 2) according to antibacterial assay (optical density, OD, left graph) and in addition for MRSA (*n* = 3) according to the more sensitive antibacterial assay (colony-forming units, CFU, right graph). Values are shown as mean ± SD. Significant changes were assessed by pairwise Student´s *t*-test (n.s., not significant; * *p* < 0.05; ** *p* < 0.01; *** *p* < 0.001).

In the high-nutrition environment, no effect of coatings on bacterial growth of *E. coli* and *S. aureus* could be observed (Figure 5A). In contrast, under low-nutrition conditions (Figure 5B), the 5%-GlcNAc-4EG (4061 ± 2184 CFU/cm^2^) and 5%-GlcNAc-6EG (22,883 ± 5172 CFU/cm^2^) modified PDMAm coatings showed a reduction in bacterial viability, compared with the untreated PETG chip (63,625 ± 13,320 CFU/cm^2^) and the unmodified PDMAm coating (60,009 ± 17,207 CFU/cm^2^).

Both the GlcNAc-2EG modified polymer and the triazole-bearing 4-hydroxymethyl derivatives (4HM-2EG, 4HM-4EG and 4HM-6EG) did not show a considerable effect compared with unmodified PDMAm, whereas the polymers 5%-GlcNAc-4EG and 5%-GlcNAc-6EG showed a significant reduction in viable MRSA and E. coli in direct contact testing under low-nutrition conditions (Figure 5B). Thus, viability was reduced by 1.2 log for the GlcNAc-4EG modified hydrogel and by 0.4 log for the GlcNAc-6EG modified hydrogel. Higher ratios of GlcNAc-4EG copolymer in the hydrogel network did not result in a stronger antibacterial effect but resulted in bacterial viabilities similar to those of 4-hydroxymethyl functionalized coatings.

**Figure 6:**
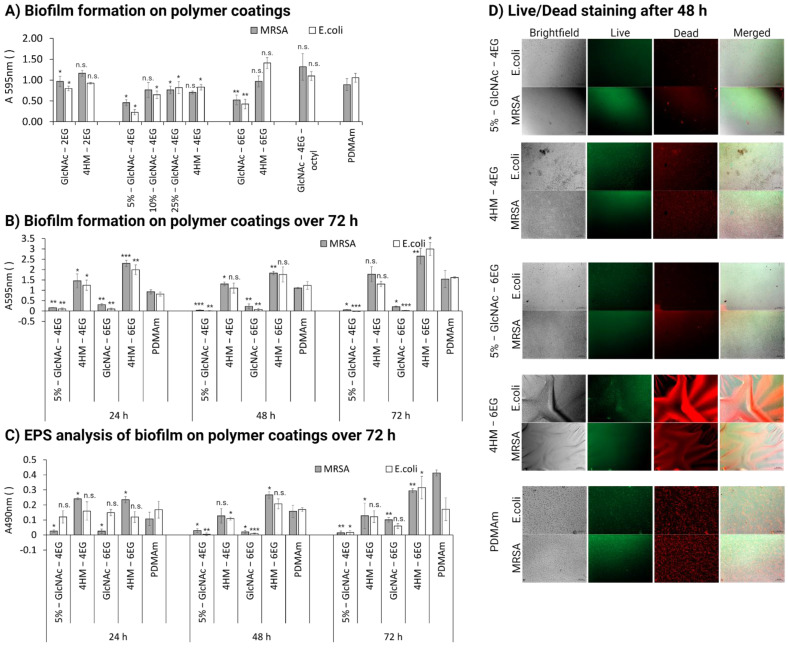
Evaluation of biofilm formation of polymer coatings. PDMAm corresponds to the unmodified acrylate coating. (**A**) Biofilm formation of *E. coli* and MRSA in the high-nutrition environment was assessed by crystal violet staining after 24 h of cultivation for all coatings as a first evaluation. (**B**) Biofilm formation of E. coli and MRSA in the high-nutrition environment was assessed by crystal violet staining after 24, 48 and 72 h of cultivation for the most active polymer coatings and their respective controls. Corresponding brightfield images are available in the supplementary materials (Figure S1) (**C**) Extracellular polymeric substance (EPS) analysis of formed biofilm by E. coli and MRSA in the high-nutrition environment was assessed by phenol-sulfuric acid method after 24, 48 and 72 h of cultivation for the most active polymer coatings and their respective controls. (**D**) Live/Dead staining of *E. coli* and MRSA in the high-nutrition environment after 24, 48 and 72 h of cultivation for the most active polymer coatings and their respective controls. Magnification is 100×; scale bar measures 0.1 mm. Values are shown as mean ± SD. Significant changes were assessed by pairwise Student´s *t*-test (n.s., not significant; * *p* < 0.05; ** *p* < 0.01; *** *p* < 0.001).

In addition to evaluating the antibacterial effect on bacterial growth, we assessed biofilm formation on the different hydrogels. The crystal violet assay for biofilm assessment showed a decrease in biofilm formation for several polymers, as shown in Figure 6A,B. In particular, the polymers 5%-GlcNAc-4EG and 5%-GlcNAc-6EG showed a significant decrease in biofilm formation compared with the unfunctionalized PDMAm coating. They showed a decrease in absorption at 595 nm for *S. aureus* of 0.46 ± 0.07 and 0.52 ± 0.12 compared with 0.89 ± 0.14, respectively. Biofilm formation for *E. coli* decreased even more: 0.23 ± 0.07 and 0.42 ± 0.11 compared with 1.06 ± 0.11. Higher GlcNAc-4EG copolymer content resulted in less biofilm inhibition (60–80% biofilm content) compared with the unmodified PDMAm hydrogel. The effect was observed over a cultivation time of 72 h (Figure 6B) and was confirmed by EPS analysis with the phenol-sulfuric acid method described by Masuko et al. (Figure 6C) [66]. In addition, we analyzed the quantity and viability of bacteria on the polymer coatings after 24, 48 and 72 h incubation, using live/dead staining (Figure 6D). Even though the live staining with DMAO in combination with the polymer coating resulted in high background noises, so that a low exposure time had to be selected and the intensity of the fluorescent stained bacteria was low, the overall effect of the polymer coatings could be confirmed. The quantity of bacteria was significantly lower for the polymers 5%-GlcNAc-4EG and 5%-GlcNAc-6EG compared to their respective controls 4HM-4EG and 4HM-6EG as can be seen in the brightfield images (Figure 6D). Furthermore, it was observed that the proportion of dead cells, especially on the 5%-GlcNAc-4EG, was higher than on the corresponding controls and particularly on the unmodified PDMAm, where a mix of dead and alive cells was visible. Additional images taken at 24, 48 and 72 h are shown in the supplementary materials (Figures S2–S4).

The inhibition of biofilm formation by GlcNAc was previously observed by Sicard et al. for different *E. coli* strains, but not for *S. aureus* [47]. However, under high-nutrition conditions, there was no longer any effect on bacterial growth observed. Antibacterial effects in the low-nutrition environment were evaluated using the OD-method to identify potential candidates and additionally evaluated for MRSA using the more sensitive CFU-method, which is more conclusive, particularly in the lower measurement range. This is attributed to the measurement procedure itself, since in the OD-method, although cells in the process of dying make up only a small part of the population, they also lead to a signal in the measurement. In contrast, only the most vital cells are taken into account in the CFU-method, as these must be able to form their own colony. The observations in the nutrient-rich environment may indicate that the polymer interferes in the metabolism of specific substrates, as studies have shown that chitosan interferes with RNA and protein synthesis [74,75,76,77]. Therefore, these substrates can no longer be used for biofilm formation or as a source of nutrients. This would explain the observed decrease in biofilm formation (high-nutrition) and bacteria viability under nutrient-poor conditions, whereas under nutrient-rich conditions, the bacteria can use other substrates as a source of nutrients, negating the growth-inhibiting effect. Another possible explanation for the lack of effect on growth under high-nutrient levels is that a higher mortality rate might have been present but was not measurable because it was obscured by significantly greater bacterial proliferation. As a result, the effect of the coatings was only observable in the low-nutrition medium, where bacterial growth was negligible and thus the increased mortality rate could be observed [78]. Nevertheless, the antibiofilm effect was observed under the nutrient-rich conditions, so the effect seems to be only partially dependent on the nutrient condition. The exact mechanism underlying the antibacterial and antibiofilm effect needs further elucidation [76].

Comparison of the different synthesized coatings showed that linker length and the amount of GlcNAc are critical parameters for the antibacterial and antibiofilm functionality of the polymers. We found that the 4EG linker yielded the best results, whereas the 2EG and 6EG linkers had decreased functionality. In addition, GlcNAc content affected the antimicrobial and antibiofilm properties: the highest effect was achieved at 5% GlcNAc and decreased with higher GlcNAc contents for the 4EG linker. This shows that the 4EG linker itself and therefore the length of the oligoethylene glycol brush affect biofilm formation and antimicrobial properties. The reason for this is most likely the steric arrangement of the functional groups and their distance from the sample surface. Depending on the distance, different interaction possibilities exist between the modified groups and the cell wall or membrane of the bacteria [79].

### 3.7. Cytotoxicity

A cytotoxicity evaluation of the polymeric coatings was carried out according to ISO 10993-5 standards using the extract method and the contact method. Extracts of the coatings were prepared according to ISO 10993-12 and 6% DMSO was chosen as the positive control for cytotoxicity. Cell viability was assessed by the MTT assay.

**Figure 7:**
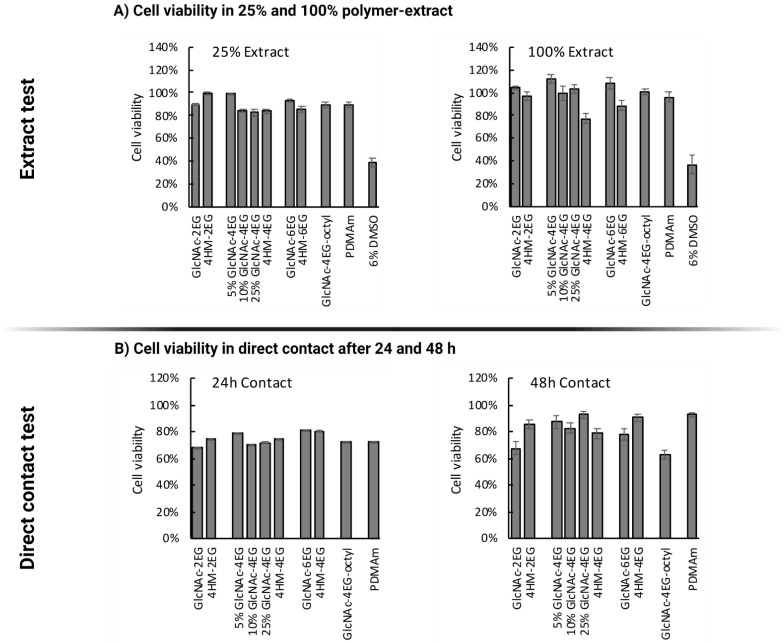
Cytotoxicity determination according to ISO 10993-5. (**A**) Cell viability measured with MTT assay after 72 h of incubation with 100% and 25% extracts; 6% DMSO was used as positive control for cytotoxicity. In both cases, no cytotoxicity was observed for the antimicrobial 5%-GlcNAc-4EG polymer. (**B**) Cell viability in direct contact test after 24 and 48 h, determined by MTT assay. The main functional polymer, 5%-GlcNAc-4EG, showed no more than a 20% decrease in cell viability by the direct contact test.

Cell viability was measured after 72 h of incubation with the prepared extracts (undiluted and diluted 4-fold with medium); results are shown in Figure 7A. No notable cytotoxic effect was observed for any GlcNAc-containing copolymer in the extract test. This included the antimicrobial and antibiofilm polymer, 5% GlcNAc-containing PDMAm hydrogel (5%-GlcNAc-4EG), whose extracts resulted in no loss of fibroblast viability. The 4-hydroxymethyl copolymers containing a tetraethylene glycol (HM-4EG) or a hexaethylene glycol linker (HM-6EG) showed minor decreases in cell viability: a 23% decrease for the 4EG linker and a 12% decrease for the 6EG linker. The extract of the 4-hydroxymethyl derivative with diethylene glycol linker (HM-2EG) showed no cytotoxicity.

For the direct contact test, the cell culture dish was directly coated with the polymers, followed by crosslinking with UV light, 3 washing steps with phosphate buffer, and seeding of cells onto the generated scaffolds. The observed cell viability after 24 and 48 h, determined by MTT assay, is shown in Figure 7B. After 24 h, cell viability was generally lower than that of the untreated cell culture dish, ranging between 60% and 80% for all polymers including the non-modified PDMAm. After 48 h, the most functional polymer against biofilms (5%-GlcNAc-4EG) and most other tested polymers showed only minor reductions in cell viability, within the range of biological systems. Only the GlcNAc-containing polymer with a diethylene glycol linker and the tetraethylene glycol octyl linker showed decreases in cell viability after 48 h (32% and 37%, respectively).

**Figure 8:**
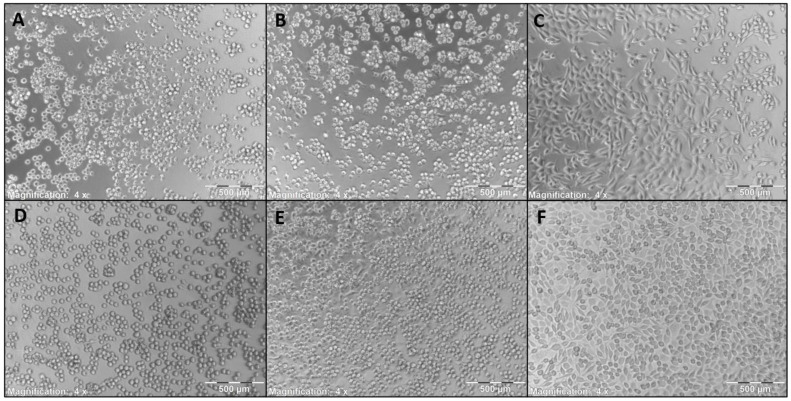
L-929 mouse fibroblasts grown on the coatings for 48 h. (**A**) GlcNAc-2EG-PDMAm, (**B**) GlcNAc-4EG-PDMAm, (**C**) 10%-GlcNAc-4EG-PDMAm, (**D**) GlcNAc-6EG-PDMAm, (**E**) PDMAm, (**F**) untreated cell culture dish. Morphology is altered to some extent, but cell viability is still given as shown via MTT test in Figure 7, in which the shown polymers show no more than 20% reduction in cell viability, so no cytotoxicity can be assumed.

Furthermore, images of the cells grown directly on the polymer coatings were taken, as shown in Figure 8. Morphology of the cells grown directly on the coatings (Figure 8A–E) is altered compared to the uncoated cell culture dish (Figure 8F). It can be observed that the cell morphology is more spheroid like, which implies a lesser adhesion. Due to the experimental conditions, where the coatings were washed after 24 or 48 h incubation prior to addition of fresh media with MTT, an adequate adherence of the cells to the coatings should be given or else the cells would have been washed away in these steps. Thereby, it can be concluded that the L-929 Fibroblasts show adherent behavior to the coatings, albeit lower than on standard cell culture plates. According to ISO 10993:5—evaluation of cytotoxicity of biomaterials, growth inhibition of >30% compared with an untreated control is considered indicative of cytotoxicity [80]. No polymers, except the GlcNAc-2EG and GlcNAc-4EG-octyl polymers showed greater growth inhibition than 30%, and therefore, all polymers except the GlcNAc-2EG and GlcNAc-4EG-octyl polymers can be considered noncytotoxic.

## 4. Conclusions

In summary, we demonstrated the successful synthesis of functionalized PDMAm hydrogel networks, suitable for polymer surface coating via UV-induced C–H insertion reaction. Stable coatings were obtained using benzophenone crosslinker chemistry. A proper sterility of the surface after UV treatment for crosslinking was shown by the absence of bacterial growth in sterile medium. The functionalized coatings showed antimicrobial and antibiofilm properties, leading to a significant reduction of microbial biofilm formation on the coated surface, for both Gram-positive (*S. aureus*) and Gram-negative (*E. coli*) bacteria. We showed up to a 1.2 log decrease in colony-forming units of the clinically relevant pathogen MRSA on surfaces treated with polymer coating. Non-cytotoxicity and biocompatibility toward fibroblast cells, evaluated according to ISO 10993-5 standards, was maintained. Overall, this work describes an interesting approach for decreasing bacterial adhesion to surfaces by selective functionalization with antiadhesive and antimicrobial molecules, preventing bacterial colonization and contamination of wound dressings or surgical implants. The use of such coatings can not only prevent many surgically induced infections or the formation of biofilms in chronic wounds but also help to accelerate wound healing by favoring fibroblasts.

## Figures and Tables

**Table 1:** Overview of the synthesized PDMAm-polymers.

Polymer	Calculated Ratio		Found Ratio (via NMR)
	MBP 14 [%] ^(a)^	11 or 12 [%] ^(b)^	MBP vs. 11/12
GlcNAc-2EG	5%	5% 11a	30:1
5%-GlcNAc-4EG	5%	5% 11b	2:1
10%-GlcNAc-4EG	5%	10% 11b	1:2
25%-GlcNAc-4EG	5%	25% 11b	1:5
50%-GlcNAc-4EG ^(c)^	5%	50% 11b	-
GlcNAc-6EG	5%	5% 11c	3:1
GlcNAc-4EG-octyl	5%	5% 11d	6:1
HM-2EG	5%	5% 12a	1:1
HM-4EG	5%	5% 12b	1:1
HM-6EG	5%	5% 12c	1:1
PDMAm ^(d)^	5%	-	-

^(a)^ 4-methacryloyloxy-benzophenone (MBP). ^(b)^ Functional GlcNAc monomers (11), Functional 4-hydroxymethyl monomers (12). ^(c)^ The 50%-GlcNAc-4EG polymer could not be obtained; instead, the reaction resulted in ester hydrolysis of the methacrylic acid ester, yielding the GlcNAc-tetraethylene glycol clickamer only. ^(d)^ The PDMAm polymer is the dimethacrylamide-co-methacryloyl benzophenone copolymer without additional functionality, serving as control for cell culture and microbiology experiments.

## Data Availability

The data presented in this study is available in the supplementary material.

## Supplementary Materials

The following are available online at https://www.mdpi.com/article/10.3390/pharmaceutics13101647/s1. Figure S1: Brightfield images after crystal violet staining of biofilm formation on polymer coatings. Figure S2: Fluorescent images after Live/Dead staining of *E. coli* and MRSA in the high-nutrition environment after 24 h of cultivation. Figure S3: Fluorescent images after Live/Dead staining of *E. coli* and MRSA in the high-nutrition environment after 48 h of cultivation. Figure S4: Fluorescent images after Live/Dead staining of *E. coli* and MRSA in the high-nutrition environment after 72 h of cultivation.
