# Supplementary material for: Methacryloyl-GlcNAc Derivatives Copolymerized with Dimethacrylamide as a Novel Antibacterial and Biocompatible Coating
Source: Pharmaceutics. 2021 Oct 9;13(10):1647. doi: 10.3390/pharmaceutics13101647 (PMC8541365; doi:10.3390/pharmaceutics13101647)
Supplement: Supplementary file 1 [file pharmaceutics-13-01647-s001.zip › pharmaceutics-1347891-supplementary-for conversion.pdf]

pharmaceutics

MDPI

Supplementary Material

Methacryloyl-GlcNAc Derivatives Copolymerized with Dimethacrylamide as a Novel Antibacterial and Biocompatible Coating

Max Borgolte [1,2], Oliver Riester [1,3,4], Tereza Kacerova [5,6], Simone Rentschler [1,3], Magnus S. Schmidt [1], Susanne Jacksch [1], Markus Egert [1], Stefan Laufer [3,4], René Csuk [2] and Hans-Peter Deigner [1,4,7,*]

1 Institute of Precision Medicine, Furtwangen University, Jakob-Kienzle Str. 17, 78054 Villingen-Schwenningen, Germany; Max.Borgolte@hs-furtwangen.de (M.B.); Oliver.Riester@hs-furtwangen.de (O.R.); S.Rentschler@hs-furtwangen.de (S.R.); Magnus.Schmidt@hs-furtwangen.de (M.S.S.); Susanne.Jacksch@hs-furtwangen.de (S.J.); Markus.Egert@hs-furtwangen.de (M.E.)
2 Department of Organic Chemistry,Martin-Luther University Halle-Wittenberg, Kurt-Mothes-Str. 2, 06120 Halle/Saale, Germany; Rene.csuk@chemie.uni-halle.de
3 Department of Pharmaceutical and Medicinal Chemistry, Institute of Pharmaceutical Sciences, Eberhard Karls University Tuebingen, Auf der Morgenstelle 8, 72076 Tübingen, Germany; Stefan.laufer@uni-tuebingen.de
4 Faculty of Science, Eberhard Karls University Tuebingen, Auf der Morgenstelle 8, 72076 Tübingen, Germany
5 Department of Chemistry, Czech University of Life Sciences, Kamýcká 129, 16500 Prague, Czech Republic; tereza.kacerova.18@ucl.ac.uk
6 Department of Chemistry, University College London, London WC1H 0AJ, UK
7 EXIM Department, Fraunhofer Institute IZI (Leipzig), Schillingallee 68, 18057 Rostock, Germany
* Correspondence: dei@hs-furtwangen.de

Citation: Borgolte, M.; Riester, O.; Kacerova, T.; Rentschler, S.; Schmidt, M.S.; Jacksch, S.; Egert, M.; Laufer, S.; Csuk, R.; Deigner, H.-P. Methacryloyl-GlcNAc Derivatives Copolymerized with Dimethacrylamide as a Novel Antibacterial and Biocompatible Coating. *Pharmaceutics* **2021**, *13*, 1647. https://doi.org/10.3390/pharmaceutics13101647

Academic Editors: Ewa Kłodzińska;, Marek Konop

Received: 3 August 2021
Accepted: 2 October 2021
Published: 9 October 2021

Publisher's Note: MDPI stays neutral with regard to jurisdictional claims in published maps and institutional affiliations.

Copyright: © 2021 by the authors. Licensee MDPI, Basel, Switzerland. This article is an open access article distributed under the terms and conditions of the Creative Commons Attribution (CC BY) license (http://creativecommons.org/licenses/by/4.0/).

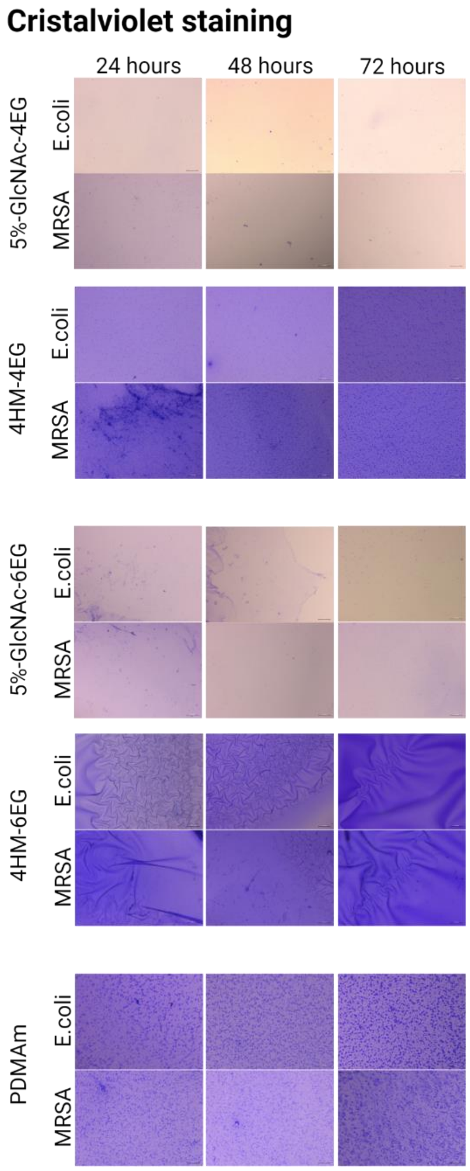

Figure S1. Brightfield images after crystal violet staining of biofilm formation on polymer coatings. PDMAm corresponds to the unmodified acrylate coating. Images were taken with 100× magnification using the microscope Observer.Z1 (Zeiss, Germany) after 24, 48 and 72 h of cultivation. Scale bar measures 0.1 mm.

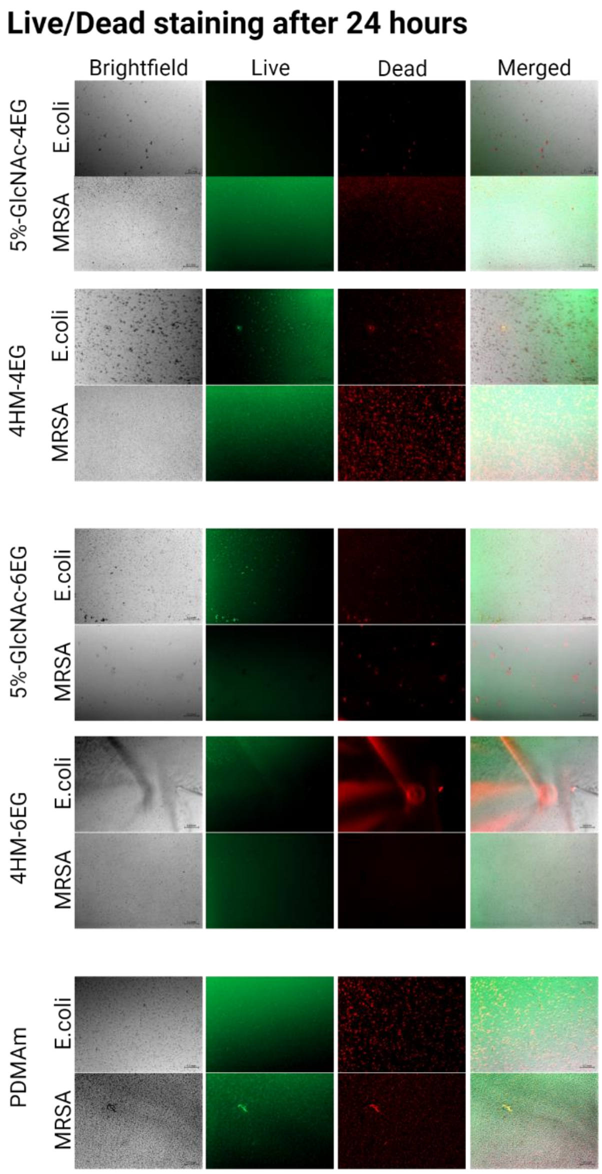

Figure S2. Fluorescent images after Live/Dead staining of *E. coli* and MRSA in the high-nutrition environment after 24 h of cultivation. PDMAm corresponds to the unmodified acrylate coating. Images were taken with 100× magnification using the microscope Observer.Z1 (Zeiss, Germany). Scale bar measures 0.1 mm.

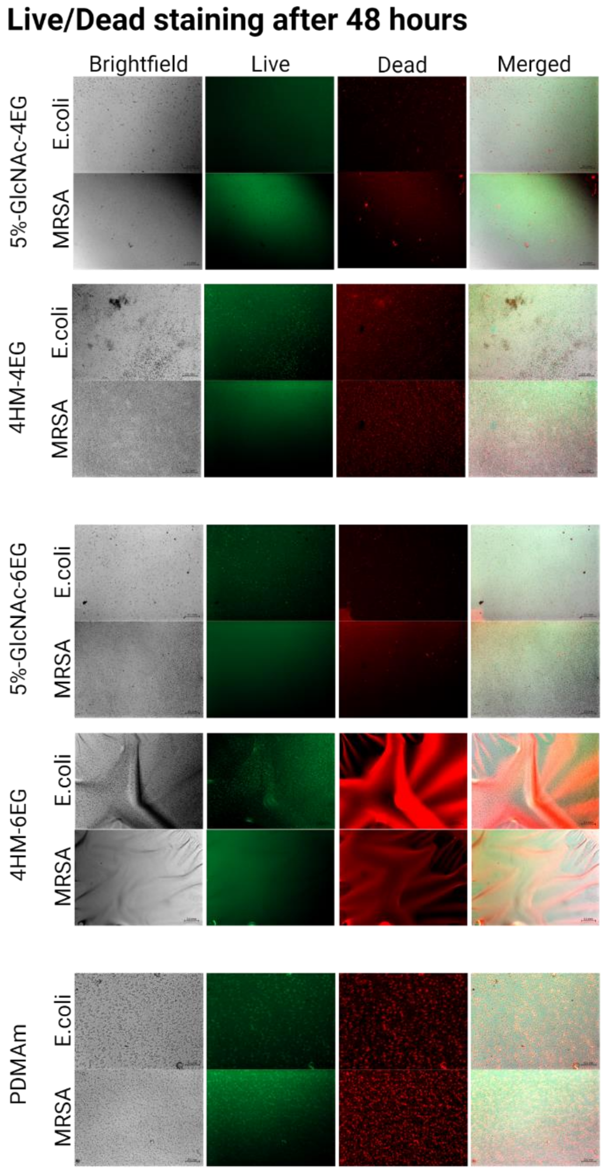

Figure S3. Fluorescent images after Live/Dead staining of E. coli and MRSA in the high-nutrition environment after 48 hours of cultivation. PDMAm corresponds to the unmodified acrylate coating. Images were taken with 100x magnification using the microscope Observer.Z1 (Zeiss, Germany). Scale bar measures 0.1 mm.

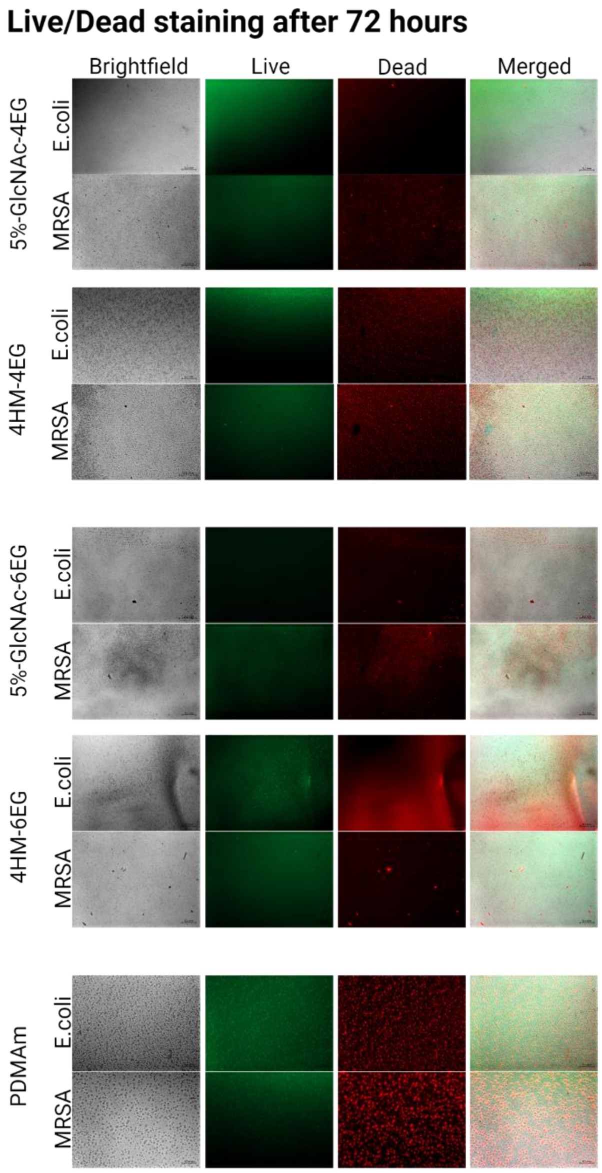

Figure S4. Fluorescent images after Live/Dead staining of *E. coli* and MRSA in the high-nutrition environment after 72 h of cultivation. PDMAm corresponds to the unmodified acrylate coating. Images were taken with 100× magnification using the microscope Observer.Z1 (Zeiss, Germany). Scale bar measures 0.1 mm.
